# Effect of Occlusal Adjustment and Subsequent Repolishing on the Translucency Parameter, Contact Angle, and Flexural Strength of Different Types of Glazed Monolithic Zirconia

**DOI:** 10.1111/jerd.70084

**Published:** 2025-12-19

**Authors:** Kelli Nunes Monteiro, Rafaela Paschoalin Nigro, Stéphanie Soares Favero, Ranulfo Benedito de Paula Miranda, Estevam Augusto Bonfante, Paulo Francisco Cesar

**Affiliations:** ^1^ Departamento de Biomateriais e Biologia Oral, Faculdade de Odontologia Universidade de São Paulo São Paulo São Paulo Brazil; ^2^ Departamento de Odontologia Restauradora, Faculdade de Odontologia Universidade Federal de Minas Gerais Belo Horizonte Minas Gerais Brazil; ^3^ Departamento de Prótese e Periodontia, Faculdade de Odontologia de Bauru Universidade de São Paulo Bauru São Paulo Brazil

**Keywords:** contact angle, flexural strength, glazed, monolithic, polishing, translucency parameter, zirconia

## Abstract

**Objectives:**

This study evaluated the effects of material and surface condition (glazed vs. polished after simulated occlusal adjustment) on translucency parameter, contact angle, and flexural strength of four monolithic zirconia.

**Materials and Methods:**

Disk‐shaped specimens (*n* = 80, Ø12 × 1 mm) were fabricated from four monolithic zirconia materials: Prettau 4 Anterior (PA), Lava Plus (LP), Cercon hT (hT), and Cercon xT (xT). All specimens were glazed; half underwent simulated occlusal adjustment followed by polishing. Translucency was measured with a spectrophotometer, contact angle with a goniometer, and flexural strength using a piston‐on‐three‐balls test. Data were analyzed via ANOVA.

**Results:**

Translucency parameter values ranged from 3.9ᶜ (PA‐Polished) to 6.9ᵃ (xT‐Glazed), while contact angle (°) values ranged from 13.4ᵈ (hT‐Glazed) to 43.7ᵃ (xT‐Polished). Flexural strength (MPa) in the glazed condition was 410.4ᵇᶜ (PA), 577.5ᵇ (LP), 576.5ᵇ (hT), and 384.5ᵇᶜ (xT); under polishing, values were 333.1ᶜ (PA), 228.8ᶜ (LP), 856.0ᵃ (hT), and 287.1ᶜ (xT).

**Conclusions:**

The xT group exhibited a higher translucency parameter compared to the LP and PA groups. Polishing increased the contact angle for all materials. Polishing subsequent to occlusal adjustment simulation preserved the flexural strength of two zirconia ceramics (PA and xT), resulted in a reduction in one (LP), and led to an increase in another (hT).

## 1 Introduction

Tetragonal zirconia polycrystal (3Y‐TZP), stabilized with 3 mol% yttrium and approximately 0.5% alumina, offers excellent mechanical properties and was developed as an alternative to metal‐ceramic restorations [1, 2]. However, its main limitation is the high rate of veneering ceramic failure when applied over 3Y‐TZP frameworks [3, 4]. To address this, monolithic zirconia was introduced. Initial attempts to improve the translucency of 3Y‐TZP involved reducing alumina content and increasing sintering temperatures, leading to only modest improvements [5]. A more effective approach was increasing yttrium oxide content beyond 4%, resulting in a higher proportion of the cubic crystalline phase, creating partially stabilized zirconia (PSZ) [6]. This microstructure enhances translucency due to the isotropic nature of cubic grains, which reduces birefringence, a phenomenon that causes light scattering [7]. However, 4Y‐PSZ and 5Y‐PSZ exhibit a reduced tetragonal phase, leading to lower mechanical properties [8].

Monolithic zirconia restorations require additional laboratory processing, such as polishing, staining, and glazing, with the glaze application helping to reduce surface roughness by adding a thin vitreous layer that fills microcracks and other defects [9, 10, 11]. Additionally, various pigments can be incorporated into the glaze layer to enhance the aesthetic outcome of dental prostheses [12, 13, 14]. However, before and/or after cementation, adjustments may be required to optimize the patient's occlusion. This process is typically performed using diamond burs, which significantly increase the surface roughness of monolithic zirconia, potentially leading to antagonist wear and biofilm accumulation [15]. On the other hand, the polishing procedure, a form of micro‐abrasion, aims to reduce the size of surface scratches [16]. Various polishing instruments are available for use in the oral cavity to promote a smooth surface, preserving natural dentition, enhancing patient comfort, and reducing biofilm formation [17, 18, 19, 20]. Numerous clinical and laboratory procedures can influence the mechanical and optical properties of monolithic zirconia. An inadequate understanding of these effects by clinicians may compromise the longevity and overall success of zirconia restorations [21, 22, 23].

When delivering glazed monolithic zirconia restorations, clinicians routinely assess occlusal contacts. Occlusal adjustments are often necessary; however, uncertainty remains regarding whether to return the restoration to the laboratory for reglazing or to perform intraoral polishing in the dental office. Although this decision is clinically relevant, the available evidence on this topic is limited. Moreover, it is important to recognize that, in many cases, intraoral adjustments may be required even after the prosthesis has been definitively cemented, throughout the lifespan of this type of restoration. Therefore, in this study, two 3Y‐TZP (Lava Plus and Cercon hT) and two 5Y‐PSZ (Prettau 4 Anterior and Cercon xT) were evaluated across two surface conditions: glazed versus polished after simulated occlusal adjustment. Therefore, the objectives were: (1) to evaluate the effect of the material and surface condition on translucency parameter (TP) of monolithic zirconia; (2) to evaluate the effect of the material and surface condition on contact angle of monolithic zirconia, and (3) to evaluate the effect of the material and surface condition on flexural strength of monolithic zirconia. The null hypotheses were as follows: (1) the material and surface condition would not affect the TP of zirconia; (2) the material and surface condition would not affect the contact angle of zirconia; and (3) the material and surface condition would not affect the flexural strength of zirconia.

## 2 Materials and Methods

A previously trained operator performed the zirconia specimens' preparations, the glaze and finishing procedures described in this section to ensure standardization. Blinding and randomization procedures were implemented during the experimental phase to minimize bias.

### 2.1 Specimens Production

Partially sintered blocks of four types of monolithic zirconia (Table 1) were transformed into cylinders with 14 mm in diameter with the help of Granulated diamond discs 220 μm (Dia‐Grid Diamond, 220 Grit Resin Bond, 8, Allied, USA) using a semi‐automatic polisher (Ecomet II, Buehler, USA). The cylinders were sectioned into 1.2‐mm‐thick specimens in a semi‐automatic cutter (Isomet 1000; Buehler) with diamond discs. After finishing in a semi‐automatic polisher with diamond sandpaper and sintering according to the recommendations of each manufacturer (Table 1) in a furnace (Zircar, USA), the specimens (*n* = 10 for each group) had approximately 12.0 mm in diameter and 1 mm in thickness.

**TABLE 1:** Materials used and sintering recommendations.

Group code	Comercial name	Manufacturer	Composition	Sintering recommendations
LP	Lava Plus	3 M ESPE	ZrO_2_; Y_2_O_3_ (> 4 to ≤ 6%); HfO_2_ (≤ 5%); Al_2_O_3_ (≤ 0.5%); others (≤ 1%)	1450°C for 2 h with heating rate of 10 °C/min
hT	Cercon hT	Dentsply Sirona	ZrO_2_; Y_2_O_3_ (5%); HfO_2_ (< 3%); Al_2_O_3_ and SiO_2_ (< 1%)	1500°C for 2 h with heating rate of 8 °C/min
PA	Prettau 4 Anterior	Zirkonzanh	ZrO_2_; Y_2_O_3_ (< 12%); Al_2_O_3_ (< 1%); SiO_2_ (< 0.02%); Fe_2_O_3_ (< 0.01%)	1500°C for 2 h with heating rate of 8 °C/min
xT	Cercon xT	Dentsply Sirona	ZrO_2_; Y_2_O_3_ (9%); HfO_2_ (< 3%); Al_2_O_3_ and SiO_2_ (< 1%)	1500°C for 2 h with heating rate of 8 °C/min

### 2.2 Glaze Application

All specimens (*n* = 80) were glazed at the surface with a thin and uniform layer (80 ± 20 μm in thickness) of Prettau Plus glaze (Zirkonzahn, Bolzano, Italy). Glaze application was performed with a brush and then the specimens were sintered in a vacuum furnace (Kerampress, Kota, Brazil) according to the manufacturer's recommendations: initial temperature, 300°C–350°C; pre‐drying time, 8 min; temperature elevation rate, 35–55 °C/min; final temperature, 750°C; and dwelling time at the final temperature, 1 min.

### 2.3 Occlusal Adjustment and Polishing

Half of the specimens (*n* = 40) underwent a simulation of occlusal adjustment with medium (grit size 90–160 μm) followed by fine‐grained (grit size 38–45 μm) diamond burs (Zir Prep FG, Jota, Ruthi, Switzerland) in a high‐speed handpiece. The next stage consisted of polishing performed with an intra‐oral polishing rubber kit (Zir Gloss, Jota, Ruthi, Switzerland) in a low‐speed handpiece (Kavo Kerr, São Paulo, Brazil). After adjustment and polishing, there was a reduction in the glaze thickness in the range of 10 to 20 μm. The reduction was measured with a caliper before and after the adjustment/polishing process. Eight groups were produced: Prettau 4 Anterior with glaze (PA‐G); Prettau 4 Anterior with glaze + adjustment + polishing (PA‐GAP); Lava Plus with glaze (LP‐G); Lava Plus with glaze + adjustment + polishing (LP‐GAP); Cercon hT with glaze (hT‐G); Cercon hT with glaze + adjustment + polishing (hT‐GAP); Cercon xT with glaze (xT‐G); and Cercon xT with glaze + adjustment + polishing (xT‐GAP).

### 2.4 Translucency Parameter and Contact Angle

The TP was measured using a CM 3700d spectrophotometer (Konica Minolta, Japan) in the visible light range (360–740 nm) and with the following standardized parameters: 10 nm intervals, observer function at 8°and D65 illuminant (daylight). This optical property defines the masking capacity of the material and was obtained by calculating the color difference of the specimens on the black and white backgrounds (*n* = 10), using the CIEDE2000 equation.

The wettability of zirconia specimens was determined by the contact angle measured using a DSA25 goniometer (Krüss, Germany) with the following standardized parameters: drop of distilled water with the volume of 1 μm^3^; 10 measurements for each specimen and sessile drop methodology. Images of the droplet were captured by a camera and the droplet profile was determined by Advance software (Kruss, Germany) and the contact angle was calculated by the Young–Laplace method.

### 2.5 Flexural Strength

The biaxial flexural strength evaluation, the specimens were fractured in a piston‐on‐three‐balls device in a Universal testing machine (EMIC DL 2000, Brazil). For each group, 10 specimens were immersed in artificial saliva at 37°C and tested at a loading rate of 0.5 mm/min. The maximum load was recorded for each specimen (N) and the flexural strength was calculated by Equation (1), according to ISO 6872/2008:

(1) $$\sigma_{f}=\frac{-0.2387\;P\;\left(X-Y\right)}{b^{2}}$$

where *P* is the fracture load, *b* is the specimen thickness, and *X* and *Y* were determined by Equations (2) and (3).

(2) $$X=\left(1+v\right)\;\ln{\left(\frac{r_{2}}{r_{3}}\right)}^{2}+\left(\frac{1-v}{2}\right)\;{\left(\frac{r_{2}}{r_{3}}\right)}^{2}$$

(3) $$Y=\left(1+v\right)\left[1+\ln{\left(\frac{r_{1}}{r_{3}}\right)}^{2}\right]+\left(1-v\right)\;{\left(\frac{r_{1}}{r_{3}}\right)}^{2}$$

where v is the Poisson's ratio, *r*_1_ is the radius of the support circle, *r*_2_ is the radius of loaded area, and *r*_3_ is the radius of specimen.

### 2.6 Data Analysis

The sample size was calculated a priori for a two‐factor factorial ANOVA (2 × 4) using an expected large effect size (Cohen's *f* = 0.40), *α* = 0.05, and 80% power, resulting in seven specimens per group. The data obtained were considered normal in relation to normality with the Shapiro–Wilk test. Data from TP, contact angle, and flexural strength of zirconia specimens were statistically evaluated by two‐way ANOVA, followed by the Tukey's test. The level of significance was set at 5% at Minitab Computer Software (Version 17, USA).

## 3 Results

### 3.1 Translucency Parameter

**FIGURE 1:**
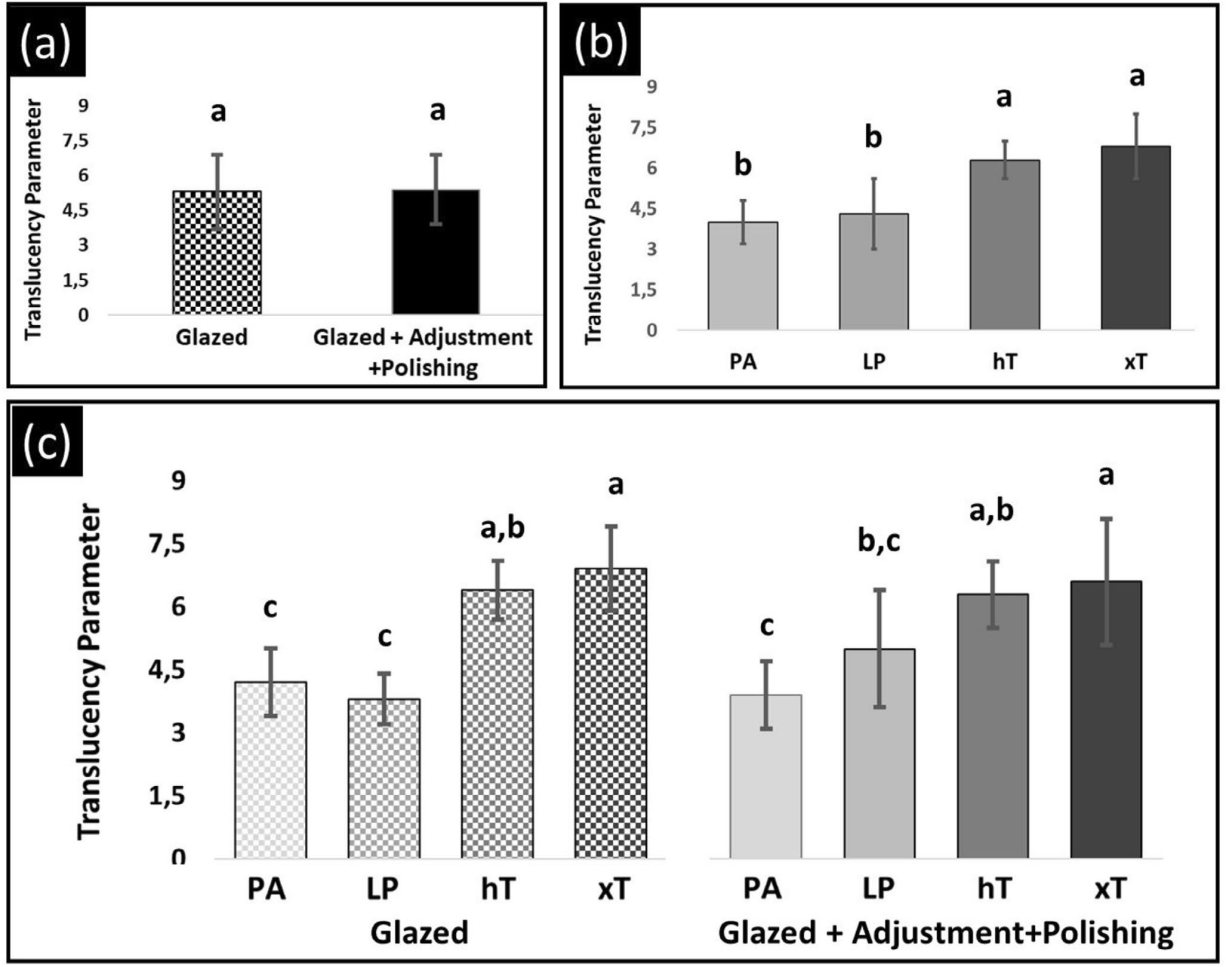
The mean translucency parameter values and their respective standard deviations are presented for each subgroup. The main effects plot illustrates the individual contributions of surface conditions (a), materials (b), and the interaction between these two factors (c) to the variation in translucency parameter. Values followed by the same letter do not differ statistically (*p* > 0.05).

Table 3 shows the mean values, standard deviations and coefficients of variation for the TP of zirconia specimens. The interaction (material versus surface condition) was statistically significant (*p* = 0.042, Figure 1 and Table 2). Table 4 demonstrates that, under the glazed surface condition, the xT (6.9) and hT (6.4) groups displayed the highest translucency values, which were statistically comparable to one another and significantly greater than those of the LP (3.8) and PA (4.2) groups. Furthermore, the LP and PA groups exhibited statistically similar translucency values in the glaze condition. After polishing, the LP group (5.0) exhibited translucency values comparable to those of the hT group (6.3). Both the hT and xT (6.6) groups, under the adjusted/polished condition, demonstrated statistically higher translucency values than the PA group (3.9). Moreover, the polishing kit was effective in maintaining the translucency of the four glazed monolithic zirconia, as the TP values for the adjusted/polished conditions of PA, LP, hT, and xT (3.9, 5.0, 6.3, and 6.6, respectively) were not significantly different from those obtained in the glazed condition (4.2, 3.8, 6.4, and 6.9).

**TABLE 2:** Summary of ANOVA of translucency parameter, contact angle, and flexural strength.

Test	Factor	df	*F*	*p*
Translucency parameter	Material	3	35.32	< 0.001
	Surface conditions	1	0.13	0.723
	Interaction: material × surface conditions	3	2.88	0.042
Contact angle	Material	3	0.95	0.423
	Surface conditions	1	158.53	< 0.001
	Interaction: material × surface conditions	3	10.96	< 0.001
Flexural strength	Material	3	10.95	< 0.001
	Surface conditions	1	3.40	0.075
	Interaction: material × surface conditions	3	10.96	< 0.001

**TABLE 3:** Mean ± standard deviation (coefficient of variation) of translucency parameter for four monolithic zirconia with two surface conditions.

Translucency parameter				
Surface conditions	Material			
	Prettau 4 Anterior	Lava Plus	Cercon hT	Cercon xT
Glazed	4.2^c^ ± 0.8 (19%)	3.8^c^ ± 0.6 (16%)	6.4^a,b^ ± 0.7 (15%)	6.9^a^ ± 1.0 (11%)
Glazed + adjustment + polishing	3.9^c^ ± 0.8 (20%)	5.0^b,c^ ± 1.4 (28%)	6.3^a,b^ ± 0.8 (22%)	6.6^a^ ± 1.5 (13%)

*Note*: Values followed by the same letter are statistically similar (*p* > 0.05).

**TABLE 4:** Mean ± standard deviation (coefficient of variation) of contact angle (°) for four monolithic zirconia with two surface conditions.

Contact angle (°)				
Surface conditions	Material			
	Prettau 4 Anterior	Lava Plus	Cercon hT	Cercon xT
Glazed	21.8^c^ ± 2.0 (9%)	22.0^c^ ± 2.1 (10%)	13.4^d^ ± 2.9 (22%)	19.7^c,d^ ± 3.2 (16%)
Glazed + adjustment + polishing	31.1^b^ ± 5.2 (17%)	37.6^a,b^ ± 10.4 (28%)	39.6^a^ ± 3.6 (9%)	43.7^a^ ± 4.9 (11%)

*Note*: Values followed by the same letter are statistically similar (*p* > 0.05).

**TABLE 5:** Mean ± standard deviation (coefficient of variation) of flexural strength (MPa) for four monolithic zirconia with two surface conditions.

Flexural strength (MPa)				
Surface conditions	Material			
	Prettau 4 Anterior	Lava Plus	Cercon hT	Cercon xT
Glazed	410.4^b,c^ ± 72.9 (18%)	577.5^b^ ± 150.9 (26%)	576.5^b^ ± 103.4 (18%)	384.5^b,c^ ± 132.1 (35%)
Glazed + adjustment + polishing	333.1^c^ ± 152.0 (45%)	228.8^c^ ± 44.2 (19%)	856.0^a^ ± 83.4 (10%)	287.1^c^ ± 171.2 (60%)

*Note*: Values followed by the same letter are statistically similar (*p* > 0.05).

### 3.2 Contact Angle

**FIGURE 2:**
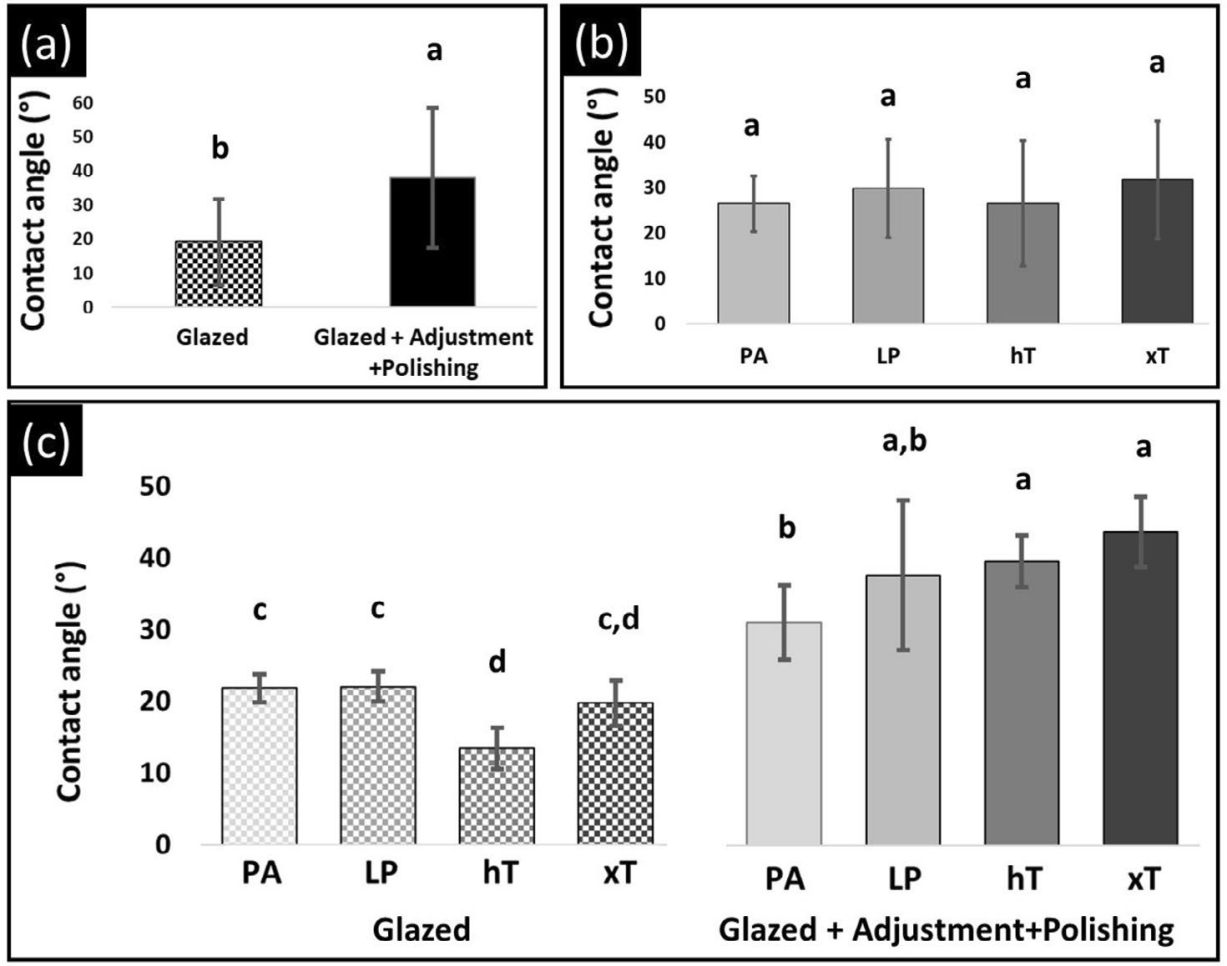
The mean contact angle values (°) and their respective standard deviations are presented for each subgroup. The main effects plot illustrates the individual contributions of surface conditions (a), materials (b), and the interaction between these two factors (c) to the variation in contact angle. Values followed by the same letter do not differ statistically (*p* > 0.05).

Table 4 shows the mean values, standard deviations and coefficients of variation for contact angle of zirconia specimens. Contact angle values ranged from 13.4° to 43.7°. Surface condition (*p* < 0.001, Figure 2 and Table 2) and the interaction (material versus surface condition) were statistically significant (*p* < 0.001, Figure 2 and Table 2). Table 5 demonstrates that, under the glazed condition, the LP (22.0°) and PA (21.8°) groups displayed the highest contact angle values, which were statistically comparable to one another and significantly greater than the hT group (13.4°). The xT group (19.7) exhibited intermediate contact angle values, which were statistically similar to those of the other three groups. In the adjusted/polished condition, the hT (39.6°) and xT (43.7°) groups exhibited the highest contact angle values, which were statistically comparable to each other and significantly higher than that of the PA group (31.1°). The surface condition affected the contact angle similarly across all materials. The adjusted/polished conditions of PA, LP, hT, and xT (31.1, 37.6, 39.6, and 43.7, respectively) exhibited higher contact angles compared to the glazed condition (21.8, 22.0, 13.4, and 19.7).

### 3.3 Flexural Strength

**FIGURE 3:**
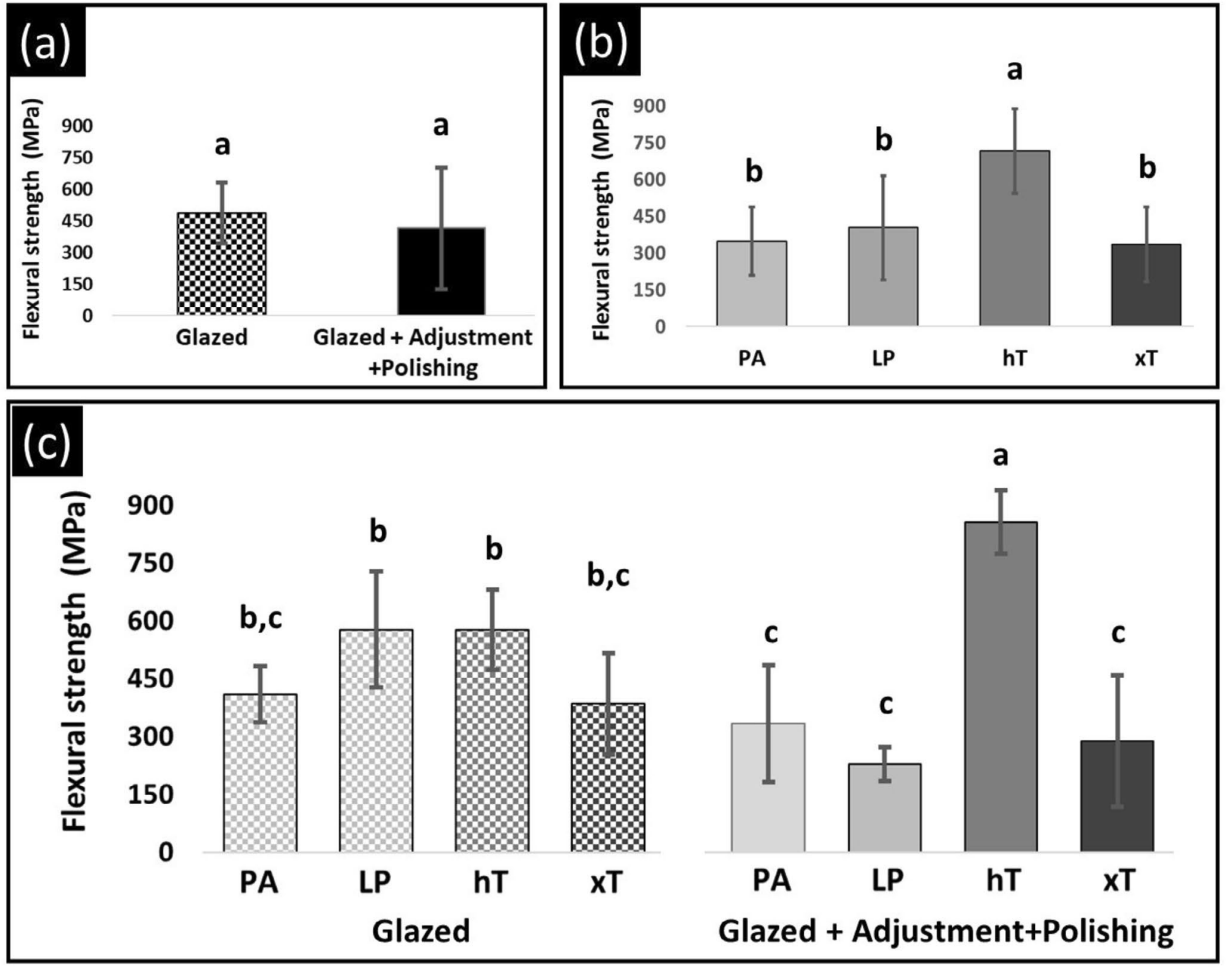
The mean flexural strength values (MPa) and their respective standard deviations are presented for each subgroup. The main effects plot illustrates the individual contributions of surface conditions (a), materials (b), and the interaction between these two factors (c) to the variation in flexural strength. Values followed by the same letter do not differ statistically (*p* > 0.05).

Table 5 shows the mean values, standard deviations, and coefficients of variation for flexural strength. Flexural strength values ranged from 287.1 to 856.0 MPa. The interaction (material versus surface condition) was statistically significant (*p* < 0.001, Figure 3 and Table 2). For the hT group, the adjusted/polished condition led to an increase in flexural strength relative to the glazed condition. Conversely, for the LP group, the adjusted/polished condition resulted in a decrease in flexural strength compared to the glazed condition. For both the PA and xT groups, the polishing kit effectively preserved the flexural strength of both zirconia materials.

## 4 Discussion

The research results showed that the xT group showed the highest numerical value of TP. This phenomenon can be attributed to the larger grain size, higher concentration of yttria (Y_2_O_3_), and the substantial presence of the cubic phase [24, 25]. The cubic phase, characterized by its non‐birefringent properties, contributes to more uniform light transmission, thereby enhancing the material's translucency [7, 26]. It is noteworthy that the polishing procedure conducted following occlusal adjustment proved effective in preserving the translucency of zirconia [27, 28]. This underscores the critical role of this clinical step in ensuring the long‐term success of monolithic zirconia restorations [29, 30]. Thus, the first null hypothesis was partially accepted, as the xT and hT groups demonstrated significantly higher TPs compared to the PA and LP groups. Additionally, the surface condition did not exert any significant effect on the TP across all four zirconia tested.

The second study hypothesis was also partially accepted, as the surface condition was found to influence the wettability of monolithic zirconia. Specifically, the polished condition increased the contact angle of all materials when compared to the glazed condition. Comparing the contact angle results for zirconia from the present study with those in the existing literature is challenging due to the wide range of factors that influence wettability, including variations in microstructure, surface roughness, material composition, and the methodology used in specimen preparation [31, 32, 33]. Consequently, discrepancies in these variables across different studies can lead to considerable differences in the reported contact angle values, making direct comparisons difficult. However, a similar trend can be observed between the present study and previous studies [33, 34], as investigations found the highest contact angle values for the polished surface condition. The literature demonstrates that polishing is an effective strategy for reducing microorganism accumulation on zirconia surfaces; however, a challenge remains in correlating wettability with biofilm formation [34, 35].

Regarding flexural strength, the material and its interaction with the surface condition influenced this property, which led to the partial acceptance of the third null hypothesis. It was observed that the hT group, when subjected to the surface adjustment/polishing condition, demonstrated higher average values in comparison to the glazed condition. In contrast, for the LP group, the surface adjustment/polishing condition led to a decrease in the mean flexural strength values relative to the glazed condition. The approximately 60% increase in resistance observed in the hT material can be attributed to the clinical adjustment process, which involves the use of diamond tips followed by polishing with diamond rubbers. This process imparts energy to the zirconia crystals through the generation of heat and friction, inducing a phase transformation [36]. As a result, the material exhibits enhanced resistance due to the development of compressive stresses [5, 6, 8]. These stresses not only contribute to increased material strength but also prevent the propagation of defects, thereby providing added protection to the material [36, 37, 38].

The adjustment simulation and subsequent polishing of the Lava Plus material probably introduced surface defects, such as scratches and microfractures. These defects are likely larger than the compression zone created, thereby compromising the material's overall strength [36, 37, 38]. It is crucial to emphasize that two simultaneous phenomena occur when 3Y‐TZP undergoes adjustment or polishing. The first is the phase transformation, which improves the mechanical properties of the material. The second is the introduction of surface defects, which may negatively affect its performance. When these defects are smaller than the compression zone generated by mechanism toughness, the material's strength increases (as observed in the hT group). However, when the defects exceed the size of the compression zone, the strength is reduced, as seen in the LP group [39, 40].

Regarding the adjusted/polished surface condition, the hT group exhibited resistance values more than twice those of the PA and xT groups. This can be attributed to the microstructure of 3Y‐TZP, which is predominantly composed of a tetragonal crystalline phase. This phase plays a critical role in the phase transformation, commonly referred to as martensitic transformation [5, 6]. This mechanism contributes to its superior flexural strength and fracture toughness relative to other dental ceramics [36]. In contrast, 5Y‐PSZ demonstrates lower fracture toughness and flexural strength, largely due to the reduced proportion of the tetragonal phase in its microstructure [5, 8]. Another contributing factor is the formation of non‐transformable tetragonal crystals, which are tetragonal grains exhibiting reduced tetragonality and a diminished ability to facilitate martensitic transformation [8, 36, 38]. The flexural strength of the xT group exhibited substantial variability, likely associated with a heterogeneous population of defects within the microstructure of the 5Y‐PSZ. These defects may have arisen from occlusal adjustment and subsequent polishing procedures.

Regarding clinical implications, polishing after occlusal adjustment is highly beneficial for restorations made from three of the four monolithic zirconia types studied. The procedure takes only a few minutes, allowing same‐appointment cementation, whereas laboratory reglazing is more time‐consuming and may disrupt chairside workflow. Surface roughness and representative scanning electron microscopy (SEM) images are important characterization parameters and have been previously published. In that study, glazed surfaces exhibited *R*_a_ values of 0.38 (PA), 0.42 (LP), 0.68 (hT), and 0.63 μm (xT), which increased after occlusal adjustment and polishing to 1.31 (PA), 1.00 (LP), 0.88 (hT), and 1.26 μm (xT) [41]. Notably, the polishing kit maintained the measured roughness only in the hT group, which also exhibited the highest flexural strength, highlighting the need for type‐specific polishing kits and standardized protocols. Although polishing of zirconia has improved, glazed surfaces generally exhibit lower roughness, which is advantageous as smoother surfaces tend to accumulate less biofilm [34].

This study has limitations that warrant consideration. First, geometric specimens were used instead of anatomical crowns. Anatomical zirconia restorations exhibit variable thicknesses across different regions, often exceeding those of the discs employed in this study, which may affect light transmission and interaction. Furthermore, finishing and polishing of anatomical restorations are more complex than for flat discs, as crown contours and grooves can restrict the contact between rotary instruments and the zirconia surface. Intraoral factors such as saliva, temperature fluctuations, and operator variability may also influence surface topography and the transformation toughening mechanism during occlusal adjustment and polishing. Second, the absence of thermocycling or mechanical aging limits the direct clinical extrapolation of these results. Third, a single glaze material was applied to all zirconia restorations. Different zirconia systems are associated with distinct materials, application protocols, and thermal cycles optimized for each system, which may affect surface properties and long‐term performance. Therefore, the findings should be interpreted with caution, and further studies using anatomical specimens and incorporating aging protocols are needed to more accurately assess the long‐term performance of zirconia restorations.

## 5 Conclusion

Based on the results of this investigation, it was possible to conclude that:

- The first null hypothesis was partially accepted, since the xT group showed a higher TP than the LP and PA groups.
- The second null hypothesis was partially accepted, since all materials tested showed lower contact angle in the glazed condition when compared with the polishing condition. In addition, in the polishing condition, the group PA showed lower contact angle than the hT and xT groups.
- The third null hypothesis was partially accepted, since for the LP group, the polishing condition resulted in lower flexural strength than the glazed condition, and for the hT group, the polishing condition resulted in higher flexural strength than the glazed condition.
- Polishing exhibits great potential as a post‐occlusal adjustment procedure for the three types of monolithic zirconia restorations evaluated (PA, hT, and xT).

## Funding

This work was supported by Conselho Nacional de Desenvolvimento Científico e Tecnológico 301205/2019‐1, 887616400/2021‐00 and Pró‐Reitoria de Pesquisa, Universidade Federal de Minas Gerais (23072.238646/2022‐33, 23072.270022/2022‐19).

## Conflicts of Interest

The authors declare no conflicts of interest.

## Data Availability

The data that support the findings of this study are available on request from the corresponding author. The data are not publicly available due to privacy or ethical restrictions.
